# Probiotics therapy show significant improvement in obesity and neurobehavioral disorders symptoms

**DOI:** 10.3389/fcimb.2023.1178399

**Published:** 2023-05-12

**Authors:** Yichen Cai, Pan Liu, Xiaolan Zhou, Jun Yuan, Qiu Chen

**Affiliations:** Department of Endocrinology, Hospital of Chengdu University of Traditional Chinese Medicine, Chengdu, China

**Keywords:** probiotics, obesity, gut-brain axis, cognitive function, microbiota

## Abstract

Obesity is a complex metabolic disease, with cognitive impairment being an essential complication. Gut microbiota differs markedly between individuals with and without obesity. The microbial–gut–brain axis is an important pathway through which metabolic factors, such as obesity, affect the brain. Probiotics have been shown to alleviate symptoms associated with obesity and neurobehavioral disorders. In this review, we evaluated previously published studies on the effectiveness of probiotic interventions in reducing cognitive impairment, depression, and anxiety associated with obesity or a high-fat diet. Most of the probiotics studied have beneficial health effects on obesity-induced cognitive impairment and anxiety. They positively affect immune regulation, the hypothalamic–pituitary–adrenal axis, hippocampal function, intestinal mucosa protection, and glucolipid metabolism regulation. Probiotics can influence changes in the composition of the gut microbiota and the ratio between various flora. However, probiotics should be used with caution, particularly in healthy individuals. Future research should further explore the mechanisms underlying the gut–brain axis, obesity, and cognitive function while overcoming the significant variation in study design and high risk of bias in the current evidence.

## 1 Introduction

Obesity has become a global epidemic, especially in the last few decades, and its prevalence is increasing at an alarming rate. Nearly 2 billion adults worldwide are considered to have overweight, with more than half of them having obesity (Ridaura et al., 2013; Hoffman et al., 2021). The impact of obesity on the quality of life is significant, as evidenced by its wide range of effects on metabolic and cognitive function (Sellbom and Gunstad, 2012; Monda et al., 2017; Agustí et al., 2018). A sizeable human cohort study reported a linear association between obesity and cognitive impairment (Farruggia and Small, 2019), which not only causes brain damage but also accelerates aging of the brain (Mattson and Arumugam, 2018). Thus, individuals with obesity may be at an increased risk of developing neurodegenerative diseases (O'Brien et al., 2017). In addition, differences in dietary habits have also been associated with cognitive performance and age-related cognitive decline (Otaegui-Arrazola et al., 2014; Proctor et al., 2017). For example, multiple studies have reported that people with obesity and animal models with diet-induced obesity show impaired learning and memory abilities (Bocarsly et al., 2015; Cordner and Tamashiro, 2015; Almeida-Suhett et al., 2017; Fazzari et al., 2018).

**Figure 1:**
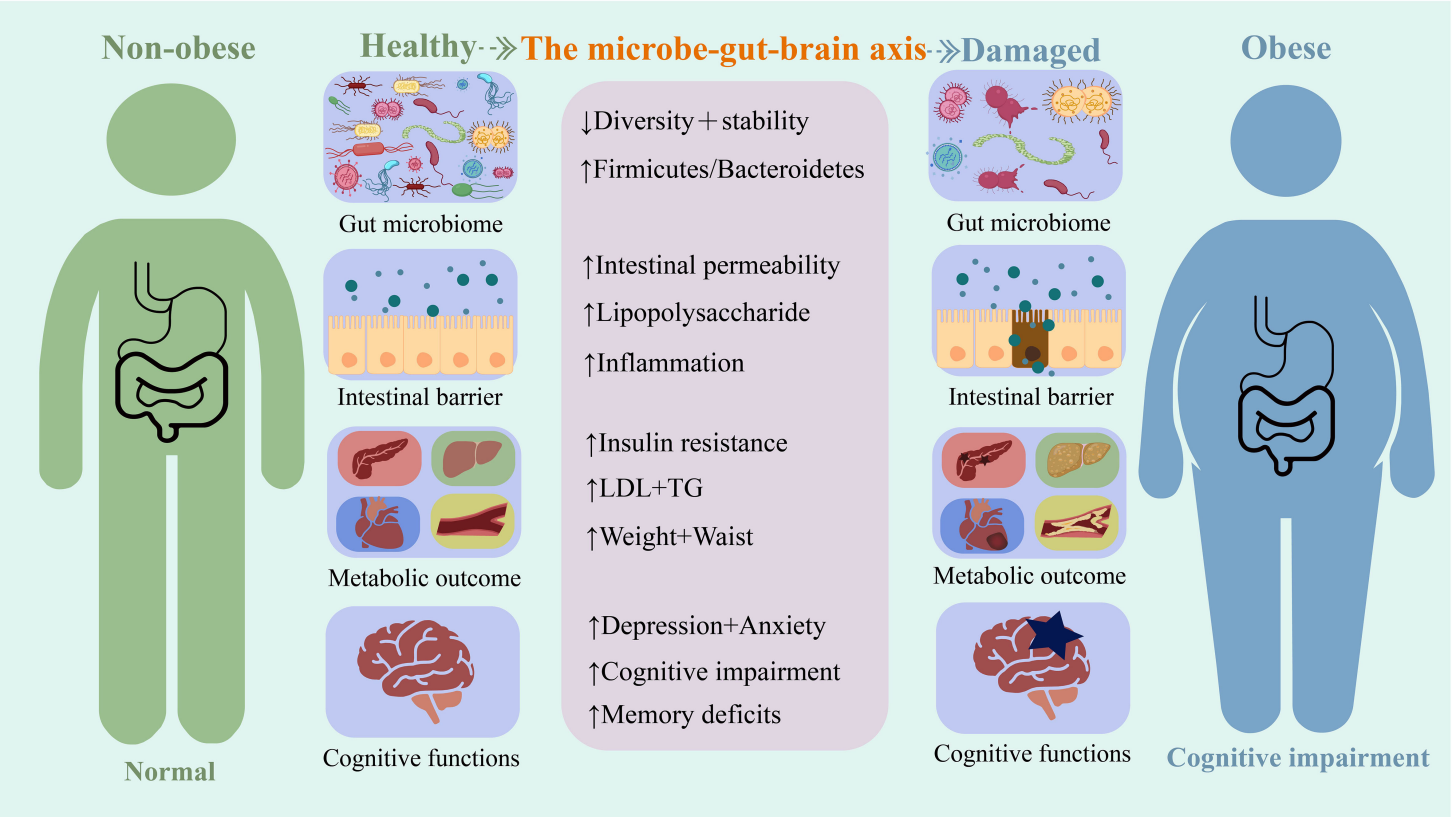
A summary of some mechanisms based on the microbial-gut-brain axis for the harm caused by obesity.

Moreover, individuals with obesity and animal models with diet-induced obesity are prone to excessive anxiety and depression-like behavior (Le Port et al., 2012; Foster and McVey Neufeld, 2013; de Noronha et al., 2017; Alonso-Caraballo et al., 2019). Obesity is frequently associated with cognitive impairment. Interactions between the gut flora and the microbial–gut–brain axis play an important role in the association observed between obesity and cognitive impairment (**Figure 1**).

Changes in the intestinal flora are closely linked to obesity. Gut microbiota composition in people with obesity differs from that in people without obesity; as such, people with obesity have gut dysbiosis (Aggarwal et al., 2013; Abenavoli et al., 2019). Dietary factors are the primary cause of obesity. Nutritional changes affect intestinal flora and cause intestinal ecological disorders to some extent. Western or high-fat diets (HFD) can cause ecological intestinal diseases (Turnbaugh et al., 2008; Hildebrandt et al., 2009; Li et al., 2009). The exact mechanisms linking intestinal ecological dysregulation and obesity are not yet fully understood. However, several possible pathways have been identified. Gut dysbiosis promotes weight gain and obesity by inducing an inflammatory response, reducing fat and cholesterol metabolism, and decreasing insulin sensitivity (Baothman et al., 2016; Rogers et al., 2016; Bliss and Whiteside, 2018). Intervention at the microbiological level using probiotics for gut ecological dysbiosis can change the composition of the gut flora (Ji et al., 2012) and thus reduce obesity-related symptoms, such as fat and cholesterol levels as well as weight gain (Ji et al., 2012; Park et al., 2013).

Additionally, gut flora affects the cognitive behavior of organisms *via* the microbial–gut–brain axis. This influence of the gut flora on the gut–brain relationship results in various mood or cognitive disorders such as anxiety, depression, and disturbances in learning and memory (Noble et al., 2017; Cryan et al., 2019). Previous studies have found that gut flora can influence cognitive behavior by affecting insulin sensitivity and inflammatory pathways; gut flora also affects obesity in the same ways (Pistell et al., 2010). This overlap could partly explain the higher susceptibility of people with obesity to cognitive deficits, anxiety, and depressive behaviors as compared to healthy people. Transplantation of gut microbiota of individuals with obesity into healthy individuals can also cause neurobehavioral changes (Bruce-Keller et al., 2015). This strongly suggests that early dietary modification and intervention centered on the microbial–gut–brain axis are noteworthy non-pharmacological therapies that may benefit both obesity and cognitive behavior.

Several metabolites produced by intestinal flora during dietary metabolism, such as trimethylamine (Vogt et al., 2018), short-chain fatty acids (Qian et al., 2021), and bile acids (MahmoudianDehkordi et al., 2019), are associated with neurodegenerative diseases. Several new therapeutic modalities, including prebiotics and probiotics, may normalize gut microbiota composition, modulate the gut–brain barrier, suppress neuroinflammation, and reduce the risk of developing neurodegenerative diseases (Green et al., 2020). As microbiota can have a wide range of effects on metabolic and cognitive behavior, probiotic interventions at the gut microbiota level are a promising approach to preventing or treating obesity-related cognitive deficits, anxiety, and depression.

The beneficial effects of probiotic interventions in obesity and metabolic diseases have been extensively reviewed (Million et al., 2012; Kobyliak et al., 2016; Azad et al., 2018). At the same time, probiotics can also improve cognitive impairment and some neurobehavioral disorders, such as anxiety, major depression, and Alzheimer’s disease (AD), all closely related to gut ecological disorders (Luna and Foster, 2015; Leblhuber et al., 2021). In previous animal and clinical studies, interventions with gut microbes using a single or a combination of probiotic strains exerted a positive effect on cognitive impairment and neurobehavioral disorders (Ansari et al., 2020). These benefits were demonstrated by an adequate reduction in cognitive impairment, depression, and anxiety in some patients after probiotic treatment, accompanied by improved communication and social skills (Zhu et al., 2021). In addition, probiotic treatment has also shown definite improvements in various disease parameters, such as specific biomarkers for diseases including amyloid beta in AD, gastrointestinal symptoms often seen in patients with major depression, and hypothalamic–pituitary–adrenal (HPA)-related stress responses in patients with anxiety states (Slyepchenko et al., 2014; Pirbaglou et al., 2016; Ng et al., 2019; Kesika et al., 2021).

**Figure 2:**
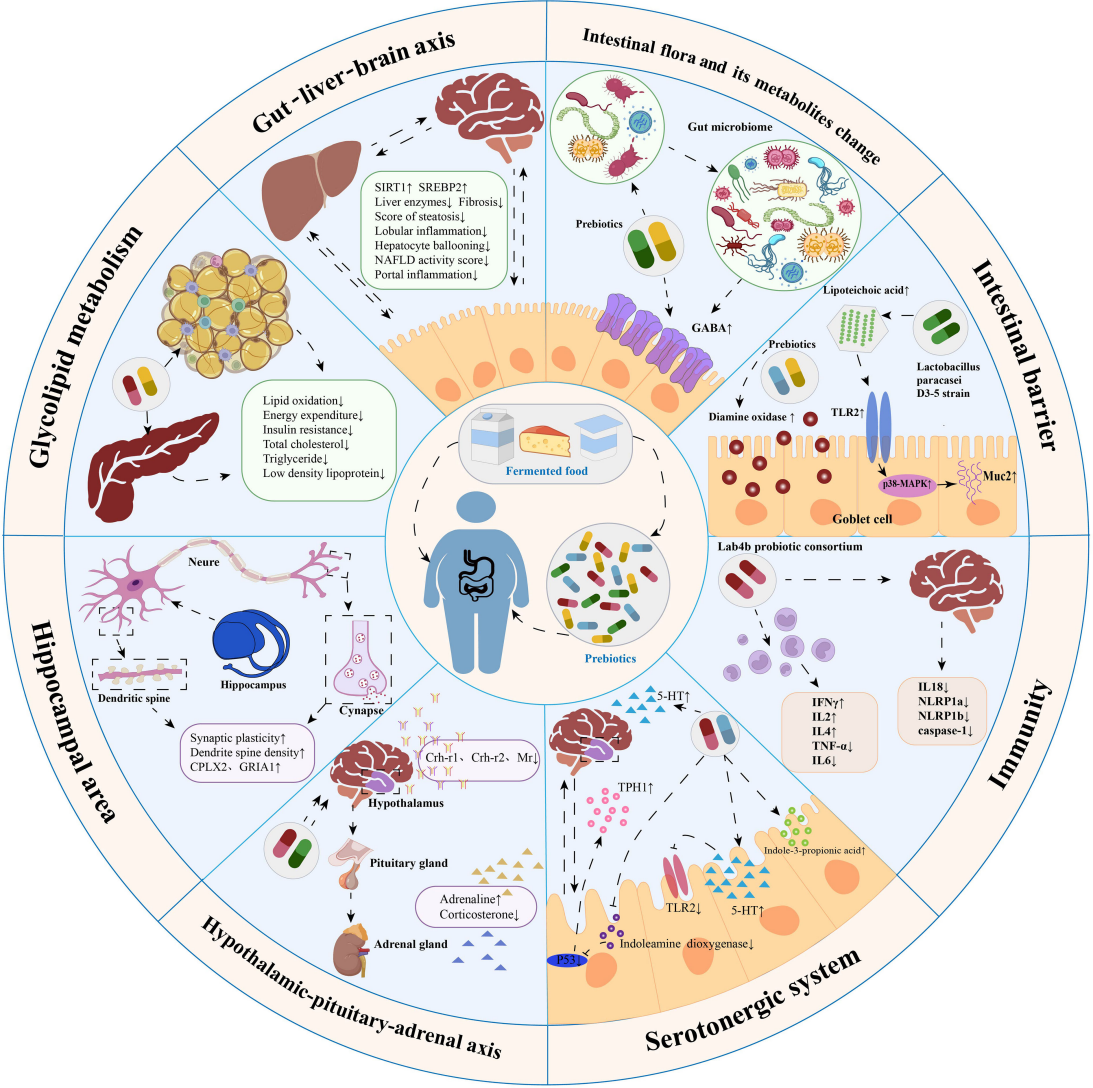
Summary of mechanisms by which probiotics modulate obesity-related cognitive impairment. Probiotic therapy can maintain intestinal barrier stability, reduce intestinal permeability, inhibit inflammatory responses, regulate intestinal microbiota composition, modulate the gut-liver-brain axis, regulate glucolipid metabolism, promote neurogenesis in the hippocampus, regulate the hypothalamic-pituitary-adrenal axis and modulate the serotonergic system. These mechanisms are essential channels for probiotics to treat obesity-related cognitive impairment.

Despite the growing body of scientific evidence on probiotic interventions in the microbial–gut–brain axis to promote weight loss and prevent cognitive dysfunction, a review of the effectiveness of probiotics in treating obesity-related neurobehavioral symptoms is yet to be published. To fill this gap in the literature, we comprehensively reviewed the effects of probiotic therapy on behavioral outcomes in populations with obesity, HFD-induced cognitive impairment, and anxiety-like and depression-like behaviors in animal studies. The data obtained from this review was then sorted on the basis of the potential mechanisms of probiotics and the microbial–gut–brain axis in the prevention and treatment of obesity-related cognitive decline disorders, as shown in **Figure 2**.

## 2 What cognitive impairment is caused by obesity?

Obesity in humans is negatively associated with cognitive performance, and individuals suffering from obesity for extended periods of time are associated with a high risk of developing cognitive impairment and dementia (O'Brien et al., 2017). Several clinical studies have explored the relationship between obesity and cognitive impairment (Morys et al., 2021; Ren et al., 2021; Anand et al., 2022; Li et al., 2022; Zhang et al., 2022). A recent cross-sectional analysis found that systemic and visceral obesity were associated with reduced cognitive scores after adjusting for cardiovascular risk factors, education level, and cerebrovascular impairment, and that strategies to prevent or reduce obesity could protect cognitive function in adults (Anand et al., 2022). A cohort study with an 8-year follow-up showed that metabolic disorders caused by obesity are potential mediators of cognitive impairment and dementia (Morys et al., 2021). The gold standard marker for obesity, the body mass index (BMI), has been positively correlated with the degree of cognitive impairment (Zhang et al., 2022). In addition to BMI, waist-to-hip ratio (WHR), fat mass (FM), and abdominal fat (AF) are markers that are strongly associated with cognitive performance in the older population (Zhang et al., 2022). Trajectory studies have shown that, in older adults with high BMI and FM, lowering WHR or AF can prevent the onset of cognitive impairment or cognitive decline (Li et al., 2022). In addition to overweight, underweight also increases the risk of cognitive impairment in older people (Ren et al., 2021). This suggests an inextricable relationship between weight and cognitive ability which is independent of sex (Guo et al., 2021).

Further evidence suggests that prenatal maternal obesity affects the neurodevelopment and cognitive function of the offspring (Liu et al., 2021). In a cross-sectional study of 778 individuals, Liu et al. (2021) found that maternal pre-pregnancy overweight and obesity were strongly associated with poor cognitive performance and social skills in children. Further studies have found that maternal transplantation of gut microbiota from donors with obesity also leads to mental and social behavioral impairment in offspring (Liu et al., 2021). Maternal obesity may impair the placenta by modulating gut microbial composition, and the microbial–gut–brain axis may be the primary mechanism by which maternal obesity leads to cognitive and social dysfunction in offspring (Liu et al., 2021). These findings suggest a strong link between obesity and cognitive impairment, which can cross gender and age and even have a genetic impact on offspring (Liu et al., 2021).

In addition to clinical evidence, the relationship between obesity and cognitive impairment has been confirmed in animal studies. Consistent with clinical data, numerous experimental studies using animal models of HFD-induced obesity have revealed multiple mechanisms of obesity-induced cognitive impairment (Shi et al., 2020). For example, obesity can cause cognitive impairment by damaging the hippocampi (Shi et al., 2020). High-fat diets reduce neurogenesis, synaptic plasticity, and neuronal growth markers in mice (Shi et al., 2020). The induction of these diets also led to altered inflammatory gene expression in the hippocampi of mice (manifested by elevated monocyte chemoattractant protein-1 [MCP-1]) and an increase in microglia while promoting premature aging of the hippocampus (Henn et al., 2022). Obesity can indirectly cause cognitive impairment through altered metabolism and dysbiosis in the intestinal environment. A HFD causes thinning of the colonic mucus layer, reduction in tight junction proteins, and an increase in intestinal permeability in mice, which further causes changes in the gut microbial composition, resulting in endotoxins entering circulation and triggering neuroinflammation (Shi et al., 2020). This suggests that gut microbial composition is influenced by obesity and HFD. This change further induces neuroinflammation through the microbial–gut–brain axis, damaging the hippocampus and leading to cognitive impairment. Therefore, it is crucial to identify the potential mechanisms underlying the role of the microbe–gut–brain axis in obesity-associated mental decline.

## 3 The role of the gut microbiome in obesity-associated cognitive impairment

The gastrointestinal tract contains a diverse population of microorganisms that maintain the intestinal barrier and promote compound metabolism. Obesity usually reduces this diversity (Le Chatelier et al., 2013) and affects cognitive function (Leigh and Morris, 2020). Therefore, post-obesity gut microbiome may serve as a reliable therapeutic target. This is reflected in the “microbe–gut–brain axis” between obesity and cognitive impairment, where alterations in the microbiome can activate neural, immune, and hormonal communication systems between the gut and brain, thereby affecting behavior and neurophysiology. However, the interplay between these mechanisms and how they affect the overall metabolic state of individuals remain to be fully understood. Therefore, discussing how the microbiome can target obesity-related cognitive impairment requires examining the unique channels mediating obesity–gut microbe–brain interactions.

### 3.1 Gut microbiome and obesity

The gut microbiota is a complex ecosystem of microbes, archaea, viruses, and their genes. It includes more than 1000 unique strains of bacteria with prokaryotic populations, exceeding the total number of human cells by orders of magnitude (Dinan and Cryan, 2017). Bacteria are vital members of the gut microbiota (Almeida et al., 2019). Macrogenome sequencing of healthy adult feces has identified Firmicutes and Bacteroidaceae as significant groups of intestinal flora (He et al., 2021). Microbial diversity is associated with the host’s health and pathological states. An increased susceptibility to obesity may occur in the presence of insufficient microbial diversity to maintain these functions or in the presence of a metabolically disordered gut microbiota. Recent studies have confirmed that the gut microbiota in individuals with obesity is significantly lower in diversity compared to that of individuals without obesity (Abenavoli et al., 2019) (Le Chatelier et al., 2013).

Although complex genetic, social, and environmental factors contribute to the development of obesity, its core cause is chronic energy imbalance. The intestinal flora can promote energy storage by inhibiting heat production in brown adipose tissue and promoting the expansion of white adipose tissue. In addition, the microbiome can dynamically respond to various internal and external physiological signals such as food intake, energy demand, and stress to maintain metabolic homeostasis. Short-chain fatty acids in the body are products of microbial activity in the gut and can facilitate the digestion of dietary fiber (Wong et al., 2006; Koh et al., 2016). Ding et al (Bäckhed et al., 2004). established an HFD mouse model in a sterile environment and identified a causal relationship between the microbiome and the obesity phenotype, with the gut microbiota influencing energy harvesting and storage in the host diet. Gut microbes assist the host in digesting difficult nutrients. Gut bacteria can degrade plant polysaccharides and complex carbohydrates into short-chain fatty acids such as butyric, propionic, and acetic acids (Turnbaugh et al., 2006). The composition and size of the short-chain-rich acid pool are essential determinants of the host metabolic state (Nehra et al., 2016).

Obesity is a generalized low-grade inflammatory state. A wide range of inflammatory markers and proinflammatory cytokines are strongly associated with the development of obesity (Bahceci et al., 2007; Choi et al., 2013). Although the causal relationship between obesity and inflammation is not fully understood, several lines of evidence suggest that an abnormal gut microbiota is a crucial regulator of immune signaling in the obesity state (Ukena et al., 2007; Telesford et al., 2015; Vallianou et al., 2019). First, lipopolysaccharides (LPS) from the cell walls of pathogenic gram-negative microorganisms in the gut can bind to Toll-like receptors (TLRs) in the mucosa and peripheral tissues, initiating a proinflammatory signaling cascade (Ukena et al., 2007). Bacteroidaceae can also promote differentiation of regulatory T cells and prevent inflammatory responses (Telesford et al., 2015). Second, intestinal flora is crucial for maintaining the integrity of the intestinal epithelium. If this function is compromised, the transit of endotoxins from the intestine into the bloodstream increases (Vallianou et al., 2019). Elevated plasma LPS levels can be detected in patients and mice with obesity, a condition known as metabolic endotoxemia (Creely et al., 2007; Cani et al., 2008; Clemente-Postigo et al., 2019). Metabolic endotoxemia causes a decrease in gram-negative (Bacteroidaceae) and gram-positive bacteria (Ondee et al., 2021; Fan et al., 2022). Animal studies have shown that endotoxemia triggers cognitive impairment *via* the gut microbiome–brain axis. The application of broad-spectrum antibiotics and beta-glucan can reduce gut microbiota shifts (a decrease in the proportion of Bacteroidaceae) and improve cognitive function (Shi et al., 2020). Mechanistic studies have shown that HFD can alter the composition of the gut microbiota and regulate the TLR4 signaling pathway. This change leads to increased plasma LPS levels and inflammatory response, thereby accelerating obesity (Kim et al., 2012).

Notably, many immune cells reside at the mucosal–luminescent interface of the gastrointestinal tract and exchange immunogenic molecules with the microbiota, thus directing the immune system to recognize potentially harmful pathogens. The microbial–microglia axis is a significant immunoreactive interaction that occurs at the gut and brain levels (Abdel-Haq et al., 2019). It is mediated by cytokines, chemokines, neuropeptides, and neurotransmitters, which can act either through the blood and lymphatic systems or on the vagus nerve and spinal cord afferent projections to the brain. Once activated, the central nervous system (CNS) microglia can release large amounts of cytokines (Pocock and Kettenmann, 2007) and chemokines and recruit monocytes from the periphery *via* tumor necrosis factor-α (TNF-α)-mediated microglial activation (D'Mello et al., 2009). In mice with HFD-induced obesity, hypothalamic microglia undergo two activations: the first with an inflammatory response and a reduction in inflammatory markers; the second is the gradual replacement of these cells with bone marrow-derived myeloid cells and the return of higher levels of inflammatory markers (Valdearcos et al., 2017). In some cases, inflammatory factors such as TNF-α in the peripheral circulation of individuals with obesity stimulate microglia to produce MCP-1/C-C motif chemokine 2 (CCL2) and brain monocytes (Kerfoot et al., 2006; D'Mello et al., 2009) and activate vagal afferent neurons to induce central inflammation (Valdearcos et al., 2017).

Bile acids (BAs) promote intestinal fat absorption and play an essential role as signaling molecules in the host-gut flora dialogue. The altered gut microbiome in the obesity phenotype is accompanied by altered BAs pool composition and metabolism, which predicts a close relationship between elevated BAs levels and metabolic diseases such as obesity (Ma and Patti, 2014). Studies have shown that HFD feeding leads to an increase in total BAs in the tissues of individuals with obesity, particularly deoxycholic acid (DCA), which is associated with the reorganization of the taxonomic microbiome in favor of the growth of strains capable of handling BAs (Lin et al., 2019). In addition to their powerful antimicrobial properties, BAs may affect the development of intestinal microorganisms and exert selective pressure on bacterial genetics through their antimicrobial activity, often referred to as “intestinal soap” (Wang et al., 2021). Earlier, researchers found that direct BA supplementation caused changes in the composition of the gut microbiome, including an increase in members of the DCA-producing Firmicutes species, a change similar to that induced by an HFD (Islam et al., 2011). Thus, the conversion of BA by the intestinal flora can cause changes in the size of the BA pool. Bile acids can cause changes in the diversity and composition of the intestinal flora, dramatically affecting host physiology.

When considering the role of gut flora in the etiology of obesity, the development of obesity-related complications can be attributed to impaired gut barrier integrity and the subsequent development of metabolic endotoxemia and consequent inflammatory responses in the presence of dysbiosis of the microbiota structure.

### 3.2 The microbe–gut–brain axis: bidirectional signaling and communication pathways

The microbial–gut–brain axis is thought to be a key regulator bridging the gap between the endocrine and nervous systems (Cryan et al., 2019). Altered intestinal flora structure and abundance of short-chain fatty acids led to cognitive impairment in preterm rats, which was observed based on significant changes reported in soluble factors associated with the microbial–gut–brain axis (5-hydroxytryptamine [5-HT], gamma-aminobutyric acid [GABA], brain-derived neurotrophic factor [BDNF], interleukin [IL]-6, and TNF-α) (Lu et al., 2022). In addition, the microbiota and brain communicate with each other through chemical signals, such as LPS, neurotransmitters, microbial metabolites (short-chain fatty acids, branched-chain amino acids, peptidoglycans, and indole derivatives), endogenous cannabinoid mediators, and N-acrylamides, to regulate the body’s energy balance (Forte et al., 2020). These molecules move between the gut and brain through anatomical pathways and are released into the blood or act on the vagus nerve, enteric nervous system, and immune system. For example, conventional colony colonization reverses altered myelin formation in germ-free mice at transcriptional and ultrastructural levels (Hoban et al., 2016). In patients with obesity, concomitant with peripheral and systemic inflammation, gut ecological dysregulation and LPS-mediated endotoxemia affect brain inflammation by enhancing the passage of circulating inflammatory factors across the blood–brain barrier (BBB) (Rhea et al., 2017), stimulating microglia *via* TLR4 (Kabouridis et al., 2015) and inhibiting vagal afferent neurons (Rolig et al., 2017).

This suggests that understanding how the altered immune status is caused by obesity-induced neuroinflammation and cognitive impairment will provide new insights into the development of probiotic interventions. Restoring gut microbiota composition and microbe–gut–brain axis signaling is a new treatment for obesity-related cognitive impairment (Torres-Fuentes et al., 2017).

### 3.3 Microbiome–brain interactions and behavior

A constant two-way communication is maintained between the gut and brain. The relationship between the host, microbiota, and microbe–gut–brain axis is critical for influencing microbial populations (Suganya and Koo, 2020). Imbalances in the gut microbiota may affect brain health, and vice versa (Cryan et al., 2020; Jena et al., 2020). An animal study observed the effects of altered gut microbial composition (Higarza et al., 2021). The absence of Lactobacillus, and increased abundance of Enterobacteriaceae, Bacteroidaceae, and Peptostreptococcaceae in rats with non-alcoholic fatty liver disease (NAFLD) exhibited cognitive deficits and reduced spatial working memory (Higarza et al., 2021). Recent clinical studies have observed significantly increased levels of Enterobacteriaceae, TLR4, LPS, and peripheral inflammatory markers in the gut of patients with post-stroke cognitive impairment (Wang et al., 2022). After transplanting fecal colonies from stroke patients into mice, researchers observed that the mice had impaired BBB integrity, microglial hyperactivation, neuronal apoptosis in the CA1 region of the hippocampus, and increased Aβ deposition in the thalamus (Wang et al., 2022). Significant interactions between gut microbiota and alpha-beta deposition have also been observed in patients with AD. This interaction may be mediated by the mitogen-activated protein kinase (MAPK) signaling pathway (Li et al., 2020).

In previous clinical studies, microbiota belonging to the Bacteroidaceae family has been associated with cognitive and neurodegenerative disorders (Carlson et al., 2018; Saji et al., 2019). One study found that infants with high levels of Bacteroidaceae in the gut at one year of age had higher cognitive abilities as compared to those of infants at two years of age (Carlson et al., 2018). In a cross-sectional study, the abundance of Bacteroidaceae was lower in the gut flora of patients with dementia than that in regular patients (Saji et al., 2019). At the species level, the abundance of Bacteroidaceae was lower in patients with cognitive impairment (Cattaneo et al., 2017). Diseases may exhibit specific gut microbial profiles on opposite sides of the spectrum. For example, a randomized controlled trial observed that a particular gut microbiota was correlated with cerebrospinal fluid biomarkers of AD, with the phylum Firmicutes positively associated with tau-p181 (Nagpal et al., 2019).

In addition to the microbiota, there are interactions between the metabolites of microorganisms and the brain. Acetate is a metabolite of gut microbes. Chronic acetate deficiency leads to impaired learning and memory in mice (Zheng et al., 2021). Exogenous acetate supplementation or fecal microbial transplantation restored hippocampal synaptophysin levels in vancomycin-treated type 1 diabetic mice, whereas vagal inhibition or vagotomy attenuated these effects (Zheng et al., 2021).

In summary, there is an inextricable interaction between gut microbiota and the brain. The microbiome can affect cognitive function, anxiety, and depression-like behaviors through community characteristics and metabolite production.

### 3.4 Microbiome-altering interventions and cognition

Interventions targeting gut microbiota are promising therapies for improving cognitive function. The use of probiotic supplements in interventional studies exploring the relationship between microbiome and cognition is a mainstream therapeutic approach. A meta-analysis showed that probiotics may improve awareness in patients with AD and mild cognitive impairment by reducing inflammatory and oxidative biomarkers (Den et al., 2020). In a mouse aging model (Yang et al., 2020), researchers observed that ProBiotic-4 (consisting of *Bifidobacterium lactis*, *Lactobacillus casei*, *Bifidobacterium bifidum*, and *Lactobacillus acidophilus*) significantly attenuated age-related disruption of the intestinal barrier and BBB and improved cognitive deficits. This may be related to probiotics acting on the microbial–gut–brain axis to inhibit the TLR4- and RIG-I-mediated nuclear factor-κB (NF-κB) signaling pathways and inflammatory responses. Probiotic supplements are currently in full clinical use. A recent randomized multicenter trial showed that *B. bifidum* BGN4 and *Bifidobacterium longum* BORI promoted psychological healing and improved cognition in older people (Kim et al., 2021). This study, which was conducted in healthy older adults, provided ample evidence of the system-wide effects of probiotics on the gut-brain. Furthermore, a clinical study, microbiome analysis identified *Prevotella ruminicola*, *Bacteroides thetaiotaomicron*, and *Bacteroides xylanisolvens* as the microbiota associated with mild cognitive impairment (Aljumaah et al., 2022).

In conclusion, there is growing evidence from preclinical models and clinical studies that interventions that affect the composition of the global microbiome and target specific bacterial species can effectively change perceptions.

## 4 Preclinical and clinical evidence for probiotics in the treatment of obesity-related cognitive impairment

There is a strong relationship between obesity, gut microbial composition, and cognitive function. Probiotics are a promising solution for dysbiosis of the gut microbiota. The World Health Organization defines probiotics as live strains of microorganisms that confer health benefits to their hosts when administered at appropriate doses. Probiotics can alter the microbial populations in the gut microbiota and regulate the function of the intestinal ecosystem, which has implications for endocrine, immune, and central nervous system health (Pelsser et al., 2017; Nishida et al., 2019). Emerging evidence suggests that probiotics have potential therapeutic effects on significant nervous system disorders such as anxiety, depression, autism, and Parkinson’s disease (Lof et al., 2022). Additionally, probiotic therapy is widely used to treat metabolic diseases, such as obesity (Borgeraas et al., 2018; Pontes et al., 2021). Non-traditional studies using the microbiome and microbial–gut–brain axis as entry points for treating obesity-related cognitive impairment have been dominated by probiotic supplements, with few studies using whole fecal transfer or antibiotic treatment (Green et al., 2020). Currently, most research is conducted on mouse models. However, the journey from the preclinical to clinical stage is rigorous and difficult to cross. Fecal transplants are challenging to achieve in humans and are associated with ethical issues. The lack of targeted antibiotic therapy results in the colonization of resistant bacteria. Overall, research describing the role of the microbial–gut–brain axis in health and disease and the potential therapeutic utility of probiotics for treating obesity-related neurodegenerative and psychiatric disorders is an exciting area.

An effective way to reorganize the intestinal flora is to take live strains of bacteria (probiotics) orally, thereby promoting probiotic integration into the intestinal ecosystem. Probiotics are mostly in the form of capsules containing the required concentrations of live bacterial cells. In modern markets, consumers have access to foods enriched with probiotic flora as functional ingredients designed to improve digestive health. Not all probiotics are created equally, and their benefits to the host are host-specific and influenced by strain concentration and type. Targeted probiotic therapy requires an enhanced understanding of microbiota-host interactions to ensure the desired metabolic outcomes. Therefore, this section discusses the preclinical and clinical evidence surrounding the use of probiotics for the treatment of obesity-related cognitive impairment. The results are presented in **Tables 1** and **2**.

**Table 1:** Metabolic alterations and cognitive changes due to obesity in the included existing studies.

Time	Species	Strain	Sex, Age at experiment week 0	Grouping	Sample sizes	Type of intervention	Effect HFD vs. Control (Metabolism)	Effect HFD vs. Control (Cognition)
2013 (Ohland et al., 2013)	Mice	129/SvEv	Sex unspecified; start experiment after weaning (3 weeks old)	ND + VEH ND + Pro HFD + VEH HFD + Pro	5/6 5/6 5/6 5/6	ND (13% fat) HFD (Western-style diet; 33% fat) VEH: PBS Pro: Lactobacillus helveticus ROO52	Weight↑ F/B ratio↑ Cecal levels of total SCFA↓	Exploratory behavior↓ Fecal corticosterone↓ Anxiety-like behavior↑
2017 (Agusti et al., 2018)	Mice	C57BL-6	Male, 7-9 weeks old	ND + VEH ND + Pro HFD + VEH HFD + Pro	10 10 10 10	ND (12.4% kcal fat) HFD (60.3% kcal fat) VEH: 10% skimmed milk Pro: Bifidobacterium pseudocatenulatum	Weight↑ Total fat mass↑ Leptin, cholesterol, triglyceride, and glucose↑ Basal corticosterone↑	Leptin receptor mRNA↓ Depressive-like behavior↑ The latency in moving from the dark box to the Anxious lightbox behavior↑ Dopamine and noradrenaline↑ 5-HT concentrations in the hypothalamus↓ TLR2 protein levels in the small intestine↑
2017 (Abildgaard et al., 2017)	Rats	Sprague-Dawley	Male, 4 weeks old	ND + VEH ND + Pro HFD + VEH HFD + Pro	10 10 10 10	ND (11 kJ% fat) HFD (60 kJ% fat) VEH: carrier matrix of maize starch, maltodextrins, and vegetable protein Pro: Ecologic Barrier	Weight↑ LPS↑	Resting time↑ Barnes maze test time↑ IL6, TNF-α↑ Trek2↓ Crh-r1, Crh-r2, 11β-hsd1↑
2018 (Jang et al., 2019)	Mice	C57BL/6	Male, 7 weeks old	ND HFD + VEH HFD + OK67 HFD + PK16 HFD + Mix	8 8 8 8 8	ND (Normal diet) HFD (High-fat diet) VEH: unspecified OK67: Lactobacillus sakei OK67 PK16: Lactobacillus sakei PK16	Weight↑ TG, TC, LPS, SREBP1c↑ Colon shortening, TNF-α, and NF-κB↑	Anxiety-like behaviors↑
2018 (Chunchai et al., 2018)	Rats	Wistar	Male, 7 days old	ND + VEH ND + Pro HFD + VEH HFD + Pro	6 6 6 6	ND (19.77% energy from fat) HFD (59.28% energy from fat) VEH: PBS Pro: Lactobacillus paracasei HII01	LPS↑ F/B ratio↑ Weight, plasma insulin level, and HOMA index↑ TC and LDL↑	IL-1, IL-6 mRNA↑ Dendritic spine density↓ Hippocampus Bax and Bcl-2↑ Morris water maze test time↑
2018 (Beilharz et al., 2018)	Rats	Sprague-Dawley	Male, 10 days old	ND + VEH ND + Low Pro ND + High Pro Caf + VEH Caf + Low Pro Caf + High Pro	10 10 10 10 10 10	ND (standard chow) Caf (cafeteria diet) VEH: unspecified Pro: VSL#3	Energy consumption↑	Memory deficits↑
2019 (Wang et al., 2020)	Mice	C57BL/6J	Male, 80-82 weeks old	HFD HFD + Pro	5-8 5-8	HFD (60% kcal fat) Pro: heat-killed Lactobacillus paracasei D3–5	Hepatic steatosis↑	Ambulatory and total motor activity↓ Anxiety↑ Morris Water Maze test time↑ Gut permeability↑ Muc2 in duodenum and ileum↓
2019 (Yang et al., 2019)	Mice	C57BL/6J	Male, 3 weeks old	ND HFD HFD + VEH HFD + AKK HFD + hk-AKK HFD + Lac	8 8 8 8 6 6	ND (standard chow) HFD (60% kcal from fat) VEH: PBS AKK ATCCBAA845 hk-AKK: heat-killed AKK Lac: Lactobacillus reuteri MM4–1A (ATCC-PTA-6475)	Weight↑ F/B ratio↑	Freezing behaviors↓ Spatial learning and memory↓ Hippocampal-dependent cognitive Function↓ LTP in the hippocampus↓ TNFα, IL-1β, and IL-6 in the hippocampus↑
2019 (Patterson et al., 2019)	Mice	C57BL/6J	Male, 8 weeks old	LFD HFD HFD + Pro1 HFD + Pro2	14 14 14 14	LFD (10% kcal fat) HFD (60% kcal fat) Pro1: Lactobacillus brevis DPC6108 Pro2: Lactobacillus brevis DSM32386	Weight↑ Blood sugar↑ Insulin sensitivity↓ Plasma cholesterol↑	Basal corticosterone↑ Open-field test time↓ Cognitive functions↓
2020 (Mohammed et al., 2020)	Rats	Wistar	Male, 7 days old	ND ND + Pro HFD + VEH HFD + Pro	6 6 6 6	ND HFD VEH: distilled water Pro: Lactobacillus Plantarum EMCC-1039	Body weight percentage↑ Liver enzymes, TC, TG and LDL↑ Score of steatosis, lobular inflammation, hepatocyte ballooning, NAFLD activity score, portal inflammation, and fibrosis↑	Morris water test↓ Hippocampal BDNF↓ Pyramidal neurons appeared shrunken↑
2020 (Myles et al., 2020)	Rats	Long-Evans	32 female, 30 male, 3 weeks old	ND + VEH ND + Pro HFD + VEH HFD + Pro	m + f = 7 + 8 8 + 8 7 + 8 8 + 8	ND (10% kcal fat) HFD (40% kcal fat) VEH: malic acid, xylitol, and maltodextrin Pro: Bifidobacterium longum R0175 and Lactobacillus helveticus R0052	Weight↑ Daily calorie consumption↓ Plasma Leptin↑	IL-1β, IL-7, GM-CSF, GRO/KC, MIP-1α and MCP-1 in male↑ Anxiety like behavior↑
2020 (Zaydi et al., 2020)	Rats	Sprague-Dawley	Male, 9 weeks old	ND HFD HFD + Statin HFD + Pro	6 6 6 6	ND (standard chow) HFD (ND + 25% animal fat) Pro: Lactobacillusplantarum DR7 Statin: Lovastatin 2mg/kg/day)	Weight↑ Telomere length ↓ Indoleamine dioxygenase↑	Morris water maze test time↑ Hippocampal apoptosis and senescence↑ Hippocampal pyramidal cell↓
2020 (Ou et al., 2020)	Mice	APPswe/PS1dE9 double-transgenic	Male, 12 weeks old	ND + APP/PS1 ND + Wet HFD + APP/PS1 HFD + Wet	10 6 10 6	ND (Normal diet) HFD (High-fat diet)	Colonic mucus cells↓ Diamine oxidase↑ Serum cholesterol and triglycerides↑ Brown adipocytes ↓	Amyloid β-protein deposition↑ Y-maze test time↑ Open-field test clearing times↓ (remain attentive↓) Standing time↓(curiosity↓)
2021 (Foroozan et al., 2021)	Mice	C57BL/6	Male, 7 ± 1 week old	ND + VEH HFD + VEH HFD + Pro	6 6 6	ND (10.60% fat) HFD (45% fat) VEH: PBS Pro: LGG (LG2055)	Weight↑ Corticosterone↑	Distance traveled↓ Corner time↑ Speed of learning↓
2021 (Higarza et al., 2021)	Rats	Sprague-Dawley	Male, 220g weighted	ND HFHC HFHC + PBS HFHC + LGG HFHC + AKK	8 8 8 8 8	ND (13% kcal fat) HFHC (65% kcal fat, 2% kcal cholesterol) LGG AKK CIP107961	Weight↑	Distinguishability of new objects↓ Object recognition impairment ↑ Latency in the retention trial↑ Spatial working memory task↓ CCO in the prefrontal cortex ↓
2022 (Webberley et al., 2022)	Mice	3xTg-AD	Male, 12 weeks old	HFD + PBS HFD + Pro	10 10	HFD (21% pork, 0.15% cholesterol) Lab4b (Lactobacillus salivarius CUL61 (NCIMB 30211), Lactobacillus paracasei CUL08 (NCIMB 30154), Bifidobacterium bifidum CUL20 (NCIMB 30153), and Bifidobacterium animalis subsp. lactis CUL34 (NCIMB 30172))	Weight↑ mRNA levels of IL-10↓ mRNA levels of IL-18, NLRP1a, NLRP1b, caspase-1↑ vLDL/LDL↑ mRNA levels of SREBP2↓	Discrimination ratio↓ Total neuronal spine density in the hippocampus↓ mRNA levels of CPLX2, GRIA1, NSMF, BDNF↓
2022 (Mahboobi et al., 2022)	Human	/	Male and female, 18-50 years old	Placebo group Intervention group	39 35	Placebo Pro: Lactobacillus rhamnosus: Bifidobacterium animalis= 1:1	LPS and Zonulin did not significantly change	Serum CRP↑ MoCA scores↓

APP/PS1, APPswe/PS1dE; CCO, Cytochrome c oxidase; CPLX2, Complexin-2; GRIA1, Glutamate receptor ionotropic AMPA type subunit 1; NSMF, Neuronal migration factor; BDNF, Brain-derived neurotrophic factor; IL-10, Interleukin 10; LDL, Low-density lipoprotein; TLR2, Toll-like receptor 2; 5-HT, 5-Hydroxytryptamine; VEH: Vehicle; HFD: High-fat diet; HFHC: High-fat high-cholesterol diet; NAFLD: Non-alcoholic fatty liver disease; NASH: Non-alcoholic steatohepatitis; ND: Normal diet; PBS: Phosphate buffer saline; Pro: Probiotic; Muc2, Mucin2; NF-κB, Nuclear factor kappa B; SREBP, Sterol regulatory element-binding protein; TC, Total cholesterol; TG, Triglyceride; TNF, Tumor necrosis factor; LPS, Lipopolysaccharide; SCFA, Short chain fatty acid; CRP, C reactive protein; MoCA, Montreal Cognitive Assessment; F/B, Firmicutes/Bacteroidetes; MIP-1α, Macrophage inflammatory protein 1-alpha; MCP-1, Monocyte chemoattractant protein-1.

**Table 2:** Metabolic changes and cognitive changes following probiotic treatment in the included available studies.

Type	Time	Models	Probiotic interventions	Intervention time	Changes in intestinal flora after probiotic treatment	Effect Pro vs. HFD (Metabolism)	Effect Pro vs. HFD (Cognition)	Microbial-gut-brain axis
Animal experiments	2013 (Ohland et al., 2013)	Obesity, Anxiety	Lactobacillus helveticus ROO52	By oral gavage Three weeks, 1 × 109 CFU/day,	F/B ratio↓	Weight↓	Exploratory behavior↑ Anxiety-like behavior↓	Probiotics - Inflammation - Anxiety
	2017 (Agusti et al., 2018)	Obesity, Mood disorders	Bifidobacterium pseudocatenulatum CECT 7765	Gavaged with 10% skimmed milk, 14 weeks, 1 × 109 CFU/day	/	Weight↓ Total fat mass↓ Leptin, cholesterol, triglyceride, and glucose↓ Basal corticosterone↓	Leptin receptor mRNA↑ Depressive-like behavior↓ The latency in moving from the dark box to the Anxious lightbox behavior↓ Dopamine and noradrenaline↓ 5-HT concentrations in the hypothalamus↑ TLR2 protein levels in the small intestine↓	Probiotic-serotonin signaling pathway-mood disorders
	2017 (Abildgaard et al., 2017)	Obesity, Depression	Mix pro (B. bifidum W23, B. lactis W52, L. acidophilus W37, L. brevis W63, L. casei W56, L. salivarius W24, Lc. Lactis W19, Lc. Lactis W58; “Ecologic Barrier,” Winclove Probiotics BV)	By oral, Five weeks, 2.5 × 109 CFU/g, 4.5 g freeze-dried powder	Indolepropionic acid and indole-acrylic acid↑	No effect on body weight and LPS	Resting time↓ IL6, TNF-α↓ Trek2↑ Mr, Crh-r1, Crh-r2, 11β-hsd1↓	Probiotics - Hippocampus HPA axis - Depression
	2018 (Beilharz et al., 2018)	Obesity, Cognitive impairment	VSL#3	By gavage in 0.3 mL of maple syrup, 6.5 weeks, Low: 2.5 × 109 CFU/day, High: 2.5 × 1010 CFU/day	Streptococcus, Lactobacillus, Butyrivibrio↑	Fat metabolism pathways (for example, α-linolenic acid metabolism, synthesis, and degradation of ketone bodies) ↓ Carbohydrate, starch, and sugar metabolism↑	Memory deficits↑	Probiotics may be detrimental to healthy subjects
	2018 (Jang et al., 2019)	Obesity, Anxiety	Pro1: Lactobacillus sakei OK67 Pro2: Lactobacillus sakei PK16	Gavaged with 1% glucose, four weeks, 2 × 109 CFU/day	Firmicutes, Proteobacteria↓ Verrucomicrobia↑ delta-Proteobacteria and Deferribacteres ↓	Weight↓ TG, TC, LPS, SREBP1c↓ Colon shortening, TNF-α, and NF-κB↓	Anxiety-like behaviors↓	Probiotics-gut flora composition-NFκB/AMPK-anxiety
	2018 (Chunchai et al., 2018)	Obesity, Cognitive impairment	Lactobacillus paracasei HII01	Via oral feeding, 12 weeks, 1 × 108 CFU/day	F/B ratio↓	LPS↓ Weight, plasma insulin level, HOMA index↓ TC and LDL↓	IL-1, IL-6 mRNA↓ Dendritic spine density↑ Hippocampus Bax and Bcl-2↓ Morris water maze test time↓	Probiotics - hippocampal plasticity - cognitive impairment
	2019 (Patterson et al., 2019)	Obesity, Cognitive impairment	Pro1: Lactobacillus brevis DPC6108 Pro2: Lactobacillus brevis DSM32386	In drinking water, 12 weeks, 1 × 1010 CFU/day	Fecal flora diversity↑ F/B ratio↑	Small intestinal content of GABA↑ Weight↓ Blood sugar↓ Insulin sensitivity↑ Plasma cholesterol↓	Basal corticosterone↓ Open-field test time↑ Cognitive functions↑	Probiotics - Intestinal GABA levels - Glucolipid metabolism - Cognitive impairment
	2019 (Yang et al., 2019)	Obesity, Cognitive impairment	Pro1: AKK (ATCC BAA845) Pro2: heat-killed AKK Lac: Lactobacillus reuteri MM4–1A (ATCC-PTA-6475)	By gavage in PBS, six weeks, 5 × 109 CFU/day	F/B ratio↓	Weight↓	Freezing behaviors↑ Spatial learning and memory↑ Hippocampal-dependent cognitive function↑ LTP in the hippocampus↑ TNFα, IL-1β, and IL-6 in the hippocampus↓	Probiotics - Microglia function in the hippocampus - Cognitive impairment
	2019 (Wang et al., 2020)	Obesity, Cognitive impairment	heat-killed Lactobacillus paracasei D3–5	In drinking water, ten weeks, 1 × 109 CFU/day	Verrucomicrobiaceae, V errucomicrobiales, V rrucomicrobiae, and Akkermansia species↑ Actinobacteria, Adlercreutzia, Coriobacteriales, and Coriobacteriia↓	Hepatic steatosis↓	Ambulatory and total motor activity↑ Anxiety↓ Morris Water Maze test time↓ Gut permeability↓ Muc2 in duodenum and ileum↑	Probiotics - Intestinal permeability and intestinal inflammation - LTA-TLR2/p38/MAPK/NF-kB - Cognitive impairment
	2020 (Ou et al., 2020)	Obesity, AD	AKK	Gavaged, six months, 5 × 109 CFU/day	/	Colonic mucus cells↑ Diamine oxidase↓ Serum cholesterol and triglycerides↓ Brown adipocytes ↑	Amyloid β-protein deposition↓ Y-maze test time↓ Open-field test clearing times↑ (remain attentive↑) Standing time↑(curiosity↑)	/
	2020 (Myles et al., 2020)	Obesity, Anxiety	Pro1: Bifidobacterium longum R0175 Pro2: Lactobacillus helveticus R0052	Via syringe feeding, seven weeks, 1 × 109 CFU/day	/	Daily calorie consumption in females↑ Daily calorie consumption in males↓	IL-1β, IL-7, GM-CSF, GRO/KC, MIP-1α and MCP-1 in male↓ Anxiety like behavior↓	/
	2020 (Mohammed et al., 2020)	Obesity, NASH	Lactobacillus Plantarum EMCC-1039	By oral gavage, Two weeks, 1.2 × 109 CFU/day	/	Body weight percentage↓ Liver enzymes, total cholesterol, TG and LDL↓ Score of steatosis, lobular inflammation, hepatocyte ballooning, NAFLD activity score, portal inflammation, and fibrosis↓	Morris water test↑ Hippocampal BDNF↑ Pyramidal neurons appeared shrunken↓	Probiotics-Intestine-Liver-Brain Axis-Hippocampal TLR4/BDNF Signaling Pathway-NASH
	2020 (Zaydi et al., 2020)	Obesity, Cognitive impairment	Lactobacillus plantarum DR7	Mixed into 1 g of a food pellet, 12 weeks, 1 × 109 CFU/day	/	Weight↓ Telomere length ↑ Indoleamine dioxygenase↓	Morris water maze test time↓ Hippocampal apoptosis and senescence↓ Hippocampal pyramidal cell↑	Probiotic-serotonin signaling pathway - cognitive impairment
	2021 (Higarza et al., 2021)	Obesity, NASH	Pro1: LGG Pro2: AKK CIP107961	Gavaged, four weeks, 1 × 1010 CFU/day	Bacterial diversity↑ Lactobacillaceae↑ Ruminococacceae↑ Enterobacteriaceae↓ Bacteroidaceae↓	Weight↓	Distinguishability of new objects↑ Object recognition impairment ↓ Latency in the retention trial↓ Spatial working memory task↑ CCO in the prefrontal cortex ↑	Probiotics - Gut Microbial Composition - CCO-NASH
	2021 (Foroozan et al., 2021)	Obesity, Cognitive impairment	LGG	By gavage, Eight weeks, 1 × 108 CFU/day	/	Weight↓ Corticosterone↓	Distance traveled↑ Corner time↓ Speed of learning↑	Probiotics - Corticosterone - Cognitive impairment
	2022 (Webberley et al., 2022)	Obesity, AD	Lab4b: Lactobacillus salivarius CUL61 (NCIMB 30211), Lactobacillus paracasei CUL08 (NCIMB 30154), Bifidobacterium bifidum CUL20 (NCIMB 30153), and Bifidobacterium animalis subsp. lactis CUL34 (NCIMB 30172)	Gavaged, 12 weeks, 5 × 108 CFU/day	Lactobacilli↓ Enterobacteria↓ Coliforms↓ Yeast↓ Enterococci↑	Weight↓ mRNA levels of IL-10↑ mRNA levels of IL-18, NLRP1a, NLRP1b, caspase-1↓ vLDL/LDL↓ mRNA levels of SREBP2↑	Discrimination ratio↑ Total neuronal spine density in the hippocampus↑ mRNA levels of CPLX2, GRIA1, NSMF, BDNF↑	Probiotics-Lipid Metabolism-Inflammation-AD
Clinical Research	2022 (Mahboobi et al., 2022)	Obesity, Depression	Mix pro (Lactobacillus rhamnosus: Bifidobacterium animalis= 1:1)	By oral, nine weeks, 1.8 × 1010 CFU/day	/	LPS and Zonulin did not significantly change.	Serum CRP↓ MoCA scores↑	/

NASH, Nonalcoholic steatohepatitis; CCO, Cytochrome c oxidase; LGG, Lacticaseibacillus rhamnosus GG; AKK, Akkermansia muciniphila; LTA, Lipoteichoic acid; AD, Alzheimer’s disease; IL-10, Interleukin 10; CPLX2, Complexin-2; GRIA1, Glutamate receptor ionotropic AMPA type subunit 1; NSMF, Neuronal migration factor; BDNF, Brain-derived neurotrophic factor; GABA, gamma-aminobutyric acid; TLR2, Toll-like receptor 2; Muc2, Mucin; NF-κB, Nuclear factor kappa B; SREBP, Sterol regulatory element-binding protein; TC, Total cholesterol; TG, Triglyceride; TNF, Tumor necrosis factor; LPS, Lipopolysaccharide; Aβ, Amyloid β-protein; NAFLD: Non-alcoholic fatty liver disease; CRP, C reactive protein; MoCA, Montreal Cognitive Assessment; MIP-1α, Macrophage inflammatory protein 1-alpha; MCP-1, Monocyte chemoattractant protein-1; CFU, Colony-forming units; NLRP1, Nucleotide-binding oligomerization domain, leucine-rich repeat and pyrin domain- containing 1; LDL, Low density lipoprotein-cholesterol; HOMA, Homeostasis model assessment; HFD, High fat diet; F/B, Firmicutes/Bacteroides.

### 4.1 Preclinical evidence for the use of probiotic therapy

Current research on the use of probiotics for the treatment of obesity-related cognitive impairment has been conducted primarily using preclinical models. In this section, we review the last three years of preclinical research on probiotics for obesity-related cognitive impairment. Notably, cognitive impairment occurred secondary to obesity in the animal experiments described in this section.

#### 4.1.1 Preclinical evidence for treatment with a single probiotic

In this part of the study, the animals were administered a single strain of probiotic bacteria. We present the results according to the type of strain used.

*Akkermansia muciniphila* (AKK) of the phylum Verrucomicrobia is the only representative human intestinal microorganism (Collado et al., 2007). Obesity-related cognitive impairment may be treated by AKK *via* its influence on weight loss and metabolic improvement. In an HFD-fed AD mouse model, AKK treatment reduced body weight (Yang et al., 2019), lowered serum cholesterol and triglycerides, and promoted brown adipocyte growth (Ou et al., 2020). In the intestine, AKK treatment enhanced the intestinal barrier by improving glucolipid metabolism in AD mice, reducing serum diamine oxidase levels, and promoting colonic mucus cell growth (Lof et al., 2022). In terms of cognitive function, AKK administration delayed the pathological changes in the brain caused by AD in the HFD-fed AD mouse model. It alleviated spatial learning and memory deficits, as evidenced by an increased completion rate of the y-maze test in mice, perhaps through a reduction in cortical Aβ levels in AD mice (Ou et al., 2020). In addition to this, AKK treatment reverses HFD-induced microglial cell proliferation in the hippocampus and specifically rescues cognitive function (Yang et al., 2019). This human intestinal microorganism is often compared with the classic probiotic *Lactobacillus rhamnosus* GG (LGG). In a high-fat, high-cholesterol diet (HFHC)-induced NAFLD rat model, treatment with both LGG and AKK CIP107961 significantly reduced the abundance of Bacteroidaceae, resulting in notable changes in the microbial composition at the phylum level (Higarza et al., 2021). Compared with LGG, AKK treatment was more helpful in restoring impaired spatial working memory. AKK reversed the decline in HFHC-related cytochrome c oxidase (CCO) activity in most brain regions that were previously affected by the HFHC diet (Higarza et al., 2021). Interestingly, neither LGG nor AKK caused any significant rearrangement of fecal microbiota. The AKK strain not only improves cognitive function but also metabolic function.

*Lactobacillus plantarum* (LP EMCC-1039) is present in many fermented foods. In a rat model of HFD-induced NAFLD, LP EMCC-1039 improved cognitive function by improving fatty liver function as evidenced by an increase in the number of viable cells, increased contractility and thickness of cone cells, and significant improvements in lipid and liver enzyme indices (Mohammed et al., 2020). *Lactobacillus plantarum* DR7 (DR7) is a probiotic isolated from fresh milk. DR7 treatment significantly reduces body weight and increases telomere length in aged rats with obesity (Zaydi et al., 2020). In a behavioral assessment of aged HFD rats, DR7 treatment reduced anxiety and enhanced memory (Zaydi et al., 2020). At the same time, DR7 decreases the expression of indoleamine dioxygenase and P53 and increased the expression of TPH1, suggesting that DR7 may play a role in serotonin signaling and oxidative senescence pathways (Zaydi et al., 2020). *Lactobacillus plantarum* JBC5 (LPJBC5) is extracted from curdled milk. LPJBC5 treatment improves learning and memory in worms while improving intestinal integrity by upregulating the expression of the intestinal tight junction protein ZOO-1 (Kumar et al., 2022). Similar to DR7, LPJBC5 treatment upregulated the expression of serotonin signaling-related genes (ser-1, mod-1, and tph-1) (Kumar et al., 2022).

*Lactobacillus paracasei* D3-5 strain (D3-5) is a dead probiotic strain isolated from the intestinal tracts of infants. The D3-5 strain improves fatty liver, regulates lipid metabolism, and improves cognitive function in mice with obesity-related cognitive impairment (Wang et al., 2020). Lipoteichoic acid (LTA) is a D3-5 cell wall component that increases mucin production (Мokrozub et al., 2015). This component suppresses inflammation and improves leaky gut by regulating the TLR2/p38/MAPK/NF-κB pathway and promoting the expression of Muc2, a major isoform of mucin abundantly expressed in the intestine (Мokrozub et al., 2015). The D3-5 strain can improve cognitive function by modulating LTA levels (Wang et al., 2020). To compare the therapeutic effects of live and dead probiotics, mice were administered *Lactobacillus sakei* OK67, *tyndallized* OK67 (tOK67), and heat-stable *L. sakei* PK16 (Jang et al., 2019). All three probiotics reduced weight and improve lipid metabolism (Jang et al., 2019). In terms of cognitive function, all three probiotics acted on the NF-κB/AMPK pathway, inhibited colonic inflammation, adjusted the ratio of Proteobacteria to Bacteroidaceae, reduced anxiety-like behavior in mice, and promoted the expression of SIRT-1 in the liver and BDNF in the hippocampus (Jang et al., 2019). Overall, the anti-obesity and anti-anxiety effects of live OK67 are more potent than those of tOK67 (Jang et al., 2019).

*Bacillus pseudocatenulatum* CECT 7765 (CECT 7765) is a probiotic isolated from healthy infants. In a mouse model of obesity with depression-like behavior, CECT 7765 treatment reduced adiposity, partially restored reduced adrenaline concentrations and 5-HT levels in the hypothalamus, and improved obesity-induced pleasure-deprived depressive behavior (reduced sucrose intake) (Agusti et al., 2018). This may be achieved by lowering the fecal corticosterone levels (Agusti et al., 2018). Additionally, CECT 7765 treatment reduces obesity-induced increases in trim intestinal TLR2 protein levels; *Bifidobacterium* does not have this effect (Agusti et al., 2018).

*Lactobacillus helveticus* is a probiotic isolated from dairy products. *Lactobacillus helveticus* treatment reduces anxiety-like behavior in mice with obesity (Ohland et al., 2013). Interestingly, no alterations in short-chain fatty acids were observed in mice receiving *L. helveticus*, suggesting that this probiotic did not alter carbohydrate metabolism (Ohland et al., 2013). Under certain dietary conditions, *L. helveticus* induces innate immunity (Ohland et al., 2013). This suggests that the diet of the host and the presence or absence of active inflammation may significantly alter the ability of probiotics to regulate the physiological functions of the host.

#### 4.1.2 Preclinical evidence for mixed probiotic therapy

In this part of the study, animals were administered probiotic drugs made from a mixture of probiotic strains in a specific ratio of two or more probiotic strains simultaneously.

The Lab4b consortium combines four strains of lactic acid bacteria and bifidobacteria- *Bifidobacterium animalis subsp. lactis* and *Bifidobacterium bifidum, Lactobacillus salivarius* and *Lactobacillus paracasei* (Michael et al., 2019). In a mouse model of HFD-induced AD, Lab4b supplementation reduced body weight by decreasing very-low-density and low-density lipoprotein (VLDL/LDL) levels and reducing neuronal loss in the CA1 region of the hippocampus, as evidenced by increased miRNA levels of complexin-2 (CPLX2), glutamate receptor ionotropic AMPA type subunit 1 (GRIA1), neuronal migration factor, and BDNF (Webberley et al., 2022). Immunologically, Lab4b did not affect immune cell composition in the brain, but increased the transcription rate of key inflammatory markers, as evidenced by decreased messenger RNA (mRNA) expression levels of IL18, NLRP1a, and caspase-1 and increased expression levels of NLRC4 in brain tissue extracts from mice (Webberley et al., 2022). Additionally, supplementation with Lab4b improved the lipid profile and regulated the expression of lipogenic genes in the liver (Webberley et al., 2022). This suggests that Lab4b can slow cognitive decline and AD pathological progression during metabolic challenges, while also providing metabolic improvements which include limiting weight gain and lowering blood lipids (Webberley et al., 2022).

*Lactobacillus brevis* DPC6108 and DSM32386 are short lactic acid bacteria that produce GABA. These two GABA-producing strains were administered to mice with HFD-induced metabolic dysfunction (Patterson et al., 2019). One study observed that probiotic treatment improved glucose metabolism and insulin sensitivity in mice, while improving depression-like behavior, basal corticosterone levels, and cognitive function (Patterson et al., 2019). This may be related to insulin metabolism and tubular GABA content.

CEREBIOME^®^ (containing *Lactobacillus helveticus* R0052 and *B. longum* R0175) is a specific formulation of a probiotic blend. CEREBIOME^®^ treatment significantly improved the anxiety behavior observed in animals with obesity (Myles et al., 2020). Interestingly, the study observed that the particular benefits of CEREBIOME^®^ treatment varied by gender (Myles et al., 2020). In female mice, CEREBIOME^®^ treatment increased leptin levels compared to the placebo and was not accompanied by weight change. In males, leptin levels did not change following CEREBIOME^®^ treatment. This suggests that using sex as a factor in the study design may be unnecessary in future probiotic-related studies. VSL#3 (Consists of three strains of bifidobacteria [*B. longum* DSM 24736, *B. infantis* DSM 24737, and *B. breve* DSM 24732], four strains of *Lactobacillus* [*L. acidophilus* DSM 24735, *L. paracasei* DSM 24733, *L. bulgaricus* DSM 24734, and *L. plantarum* DSM 24730], and one strain of *Streptococcus salivarius* subsp. *thermophilus* DSM 24731) is a probiotic blend. VSL#3 treatment increased the levels of specific taxa in the gut of cafeteria diet-fed rats, such as *Streptococcus*, *Lactobacillus*, and *Butyrivibrio* (Beilharz et al., 2018). However, the administration of VSL#3 did not affect metabolic outcomes, including weight and fat levels (Beilharz et al., 2018). This suggests that probiotics are beneficial for gut ecological disorders in which memory deficits are evident, but may be detrimental in healthy subjects (Beilharz et al., 2018). These results support the development of bespoke probiotics that may be more effective in reorganizing the gut flora in a healthy direction (Beilharz et al., 2018).

In addition to previously named probiotic mixes, some studies have used unnamed probiotic mixes. Abildgaard et al. (2017) used a mixture of probiotics (consisting of eight strains: *B. bifidum* W23, *B. lactis* W52, *L. acidophilus* W37, *L. brevis* W63, *L. casei* W56, *L. salivarius* W24, and *Lc. lactis* W19, *Lc. Lactis* W58) and observed reduced hippocampal transcript levels of HPA axis-related regulators (CRH-R1, CRH-R2, and MR) in HFD-fed mice, whereas HFD alone increased these levels. Metabolomic analysis revealed that the probiotic mix increased the number of a potential neuroprotective agent (indole-3-propionic acid) (Abildgaard et al., 2017). Zeltser et al. (2020) used a mixture of probiotics (consisting of *Pediococcus acidilactici* and *pentosaceus*, *Lactobacillus plantarum*, and *Bacillus amyloliquefaciens*) to treat NAFLD in Iberian pigs. Studies have shown that feeding probiotics to pigs may impair its cognitive function and increase the levels of essential myelin proteins. It is possible that the translocation of probiotics to the intestinal mucosa caused systemic damage in young pigs (Zeltser et al., 2020).

In existing preclinical models, probiotic interventions affect the metabolism and cognition of experimental animals. These probiotics need to be further studied in humans to determine their safety and whether they will benefit target patients.

### 4.2 Clinical evidence of treatment with probiotics

Although probiotic-rich fermented foods and dairy products have been introduced into human life, clinical studies on probiotic interventions for patients with obesity-related cognitive impairment are relatively limited. There is a bidirectional relationship between obesity and neuropsychiatric states (Foroozan et al., 2021), which constitutes a subtype of the disease with a unique pathophysiological mechanism called “metabolic-mood-syndrome” (MMS) (Vogelzangs et al., 2011; de Melo et al., 2017). In a clinical study that included 74 patients with MMS, patients were randomized to consume Probio-Tec^®^ BG-VCap-6.5 (containing *L. rhamnosus* [LGG^®^] and *Bifidobacterium animalis subsp. lactis* *BB-12* ^®^) *(* Mahboobi et al., 2022). The combination of probiotics and magnesium reduced CRP levels and suppressed inflammation after nine weeks of treatment. However, the probiotics did not exert any effect on mood, cognition, or gut integrity in individuals with obesity and depression (Mahboobi et al., 2022).

The transition from preclinical to clinical trials is the end of probiotic therapy, and more clinical trials should be conducted to verify the safety and therapeutic efficacy of probiotics.

## 5 Mechanism of action of probiotic intervention in the microbial–intestinal–brain axis

Nearly three years of preclinical and clinical evidence have confirmed that the microbial–gut–brain axis bridges the regulation of obesity-related cognitive impairment by probiotics. In addition to focusing on the therapeutic effects of probiotics, it is essential to understand the specific mechanisms by which probiotics intervene in the microbial–intestinal–brain axis.

### 5.1 Intestinal permeability and intestinal inflammation

An essential feature of obesity is alteration of the intestinal flora (Turnbaugh et al., 2006) and destruction of the intestinal barrier (Cani et al., 2008; Cani et al., 2009). There is clear evidence that intestinal flora affects systemic metabolism by influencing energy balance (Cani et al., 2012), intestinal permeability (Cani et al., 2009; Muccioli et al., 2010), metabolic endotoxemia (Cani et al., 2007), and metabolic inflammation associated with obesity and related diseases (Turnbaugh et al., 2006). Disturbances in the intestinal environment can induce cognitive impairment *via* bidirectional gut-brain communication, many of which is mediated by metabolites or immune factors produced by intestinal microbes (Doifode et al., 2021). In the animal experiments mentioned earlier, obesity-related cognitive impairment was often accompanied by increased intestinal permeability and inflammation (Jang et al., 2019). Therefore, the gut is a critical node in the microbe–gut–brain axis. Many probiotics use the gut as an entry point to improve metabolic and cognitive functions in animals with obesity by maintaining gut integrity, inhibiting inflammatory factors, and modulating the microbe–gut–brain axis (Мokrozub et al., 2015; Jang et al., 2019; Ou et al., 2020).

In the intestine, there is a physical barrier between the lumen, lamina propria, and mucosa-associated lymphoid tissue, which is comprised of epithelial cells. Therefore, disruption of the intestinal epithelial barrier caused by obesity can lead to the entry of intestinal secretions and LPS into the bloodstream, further inducing an inflammatory response (Kumar et al., 2018; Xu et al., 2018). Diamine oxidase (DAO) is a highly reactive intracellular enzyme that reflects the integrity and extent of damage to the intestinal mechanical barrier and is closely associated with intestinal mucosal cells. The AKK strain normalizes the composition of intestinal flora, which is associated with an improved metabolic profile. AKK supplementation improves cognitive function by lowering serum DAO levels, protecting colonic mucus cells, reducing intestinal permeability, and inhibiting LPS entry into circulation (Ou et al., 2020). Supplementation with D3-5 can improve cognitive function by increasing LTA levels in the gut to regulate the TLR2/p38/MAPK/NF-kB pathway, promote Muc2 expression and inhibit inflammatory factors (Мokrozub et al., 2015). Supplementation with LPJBC5 upregulates the expression of the tight junction protein ZOO-1, which is closely associated with inflammatory factors and improves cognitive function (Kumar et al., 2022). Therefore, by regulating the “microbial–gut–brain axis,” probiotics can reverse HFD-induced metabolic disorders and promote the secretion of intestinal mucus protein-related molecules, thereby reducing intestinal permeability, inhibiting intestinal inflammation, and ultimately improving cognitive function.

### 5.2 Metabolic alterations

The most crucial trigger for the development of obesity-related cognitive impairment is metabolic disturbances caused by obesity. Probiotics can improve the metabolic status and reduce body weight by producing a variety of metabolites, such as short-chain fatty acids (SCFAs), which affect the microbe–gut–brain axis and improve cognitive function. Increased levels of *Bacteroides* and *Bilophila* in the gut were observed in a swine model of HFHC-induced NAFLD, leading to increased synthesis of microbial-derived bile acids and hydrogen sulfide, impaired gut barrier function, and cognitive impairment (Zeltser et al., 2020). This suggests that the gut–liver–brain axis may play an important role in obesity-related cognitive impairment (Zeltser et al., 2020). Supplementation with LP EMCC-1039 may improve NAFLD and cognitive impairment by affecting the intestine–liver–brain axis and hippocampal TLR4/BDNF signaling pathway (Mohammed et al., 2020). The intestinal flora metabolite, GABA, regulates the metabolic pattern of the host. Supplementation with GABA-producing *Lactobacillus* increases endogenous GABA concentration in the small intestine. Supplementation with *L. brevis* DPC6108 or DSM32386 improves HFD-induced glucose intolerance and impairs insulin sensitivity, depression-like behavior, basal corticosterone levels, and cognitive function in mice (Patterson et al., 2019). This may be related to probiotic interventions that increase luminal GABA content in the small intestine (Patterson et al., 2019).

Thus, probiotics can influence the microbial–gut–brain axis by modulating the metabolites of the intestinal flora and regulating glucolipid metabolism, thus exerting an effect on improving cognitive function.

### 5.3 The neuroendocrine system

The brain is one of the critical nodes of the microbe–gut–brain axis and the leading site for cognitive function formation. An imbalance in the F/B ratio of the gut flora can lead to abnormal host metabolism, disruption of serotonin signaling pathways, and further impairment of cognitive function (Liang et al., 2020; Ni et al., 2021). Probiotics can influence the microbial–gut–brain axis to improve cognitive function by modulating the structure of the intestinal flora and promoting the secretion of hormones and neurotransmitters.

The hippocampal region of the brain is responsible for the learning and memory capacities of living organisms. In animals with obesity-related cognitive impairment, neuronal damage can be observed in the hippocampal region, with reduced synapses and hippocampal plasticity accompanied by overactivation of microglia (Chunchai et al., 2018; Yang et al., 2019). Supplementation with *L. paracasei* HII01 increases the density of dendritic spines in the hippocampus. It inhibits hippocampal apoptosis and mitochondrial brain dysfunction, thereby increasing hippocampal plasticity and improving cognitive function in rats with obesity (Chunchai et al., 2018). Supplementation with *L. paracasei* HII01 results in inhibition of microglial overactivation through signaling pathways involved in neuroglial cell communication (Chunchai et al., 2018). Similarly, AKK supplementation promotes the formation of CA1 pyramidal neurons in the hippocampal region of newborn mice and improves synaptic plasticity in the hippocampal region, thereby improving cognitive function in mice with obesity (Yang et al., 2019).

Previous studies have shown an interaction between gut flora and the HPA axis (Desbonnet et al., 2008). Probiotic treatment significantly alters hippocampal transcript levels of CRH-R1 and CRH-R2 associated with HPA axis feedback (Abildgaard et al., 2017). Increased CRH-R1 signaling is significantly associated with stress and depression (Dale et al., 2015). Corticosterone is the primary hormone produced by the adrenal cortex in response to hypothalamic-pituitary stimulation. Under pressure, corticosterone concentrations were higher in the feces of mice with obesity than in those of normal mice. Supplementation with CECT 7765 reduces the fecal corticosterone concentration in mice and improves the stress response caused by obesity (Agusti et al., 2018). Similarly, LGG supplementation may reduce anxiety-related behavior by lowering serum corticosterone levels (Foroozan et al., 2021).

Serotonin signaling pathways are essential for emotional and cognitive processes (Dale et al., 2016). Ninety percent of 5-HT in the body is distributed in the intestinal canal and basal plane, with the remainder being widely distributed in the brain tissue, digestive system, and peripheral blood. It is known that 5-HT levels are high in the cerebral cortex and synapses and may act as a bridge between the gut and brain. Supplementation with CECT 7765 ameliorates obesity-induced stress by reducing 5-HT levels in the hippocampus and intestines of anxious mice (Agusti et al., 2018). Supplementation with DR7 reduces anxiety and enhances memory capacity by decreasing indoleamine dioxygenase and P53 expression and increasing TPH1 expression (Zaydi et al., 2020). DR7 may play a role in the serotonin and oxidative senescence pathways (Zaydi et al., 2020).

Thus, probiotics can influence the microbial–gut–brain axis by modulating the HPA axis and serotonin signaling pathways, promoting the secretion of hormones and neurotransmitters, and exerting an effect on improving cognitive function.

## 6 Summary and outlook

The critical roles of the gut microbiota and the microbe–gut–brain axis in obesity-related cognitive impairment have been extensively studied and validated. Through combination, we know that the inflammatory response, abnormalities in the HPA axis, and altered intestinal permeability in people with obesity are all essential mechanisms that contribute to cognitive impairment, and that the gut microbiota is intimately involved in these mechanisms. The microbial–gut–brain axis links the gut microbiota in close crosstalk with organ tissues. Probiotic therapy targets the gut microbiota and the microbe–gut–brain axis and has shown significant improvement in symptoms associated with obesity and neurobehavioral disorders.

To summarize the current animal experiments and clinical trials involving probiotics, it is known that (i) probiotics are effective in reducing body weight, lowering blood lipids, improving metabolism, and improving neurocognitive function; (ii) probiotics can target changes in the composition of the gut microbiota and the ratio between flora, depending on the type of probiotic. Still, it is worth noting that the regulated use of probiotics does not cause significant rearrangement of the gut microbiota; (iii) Several mechanisms of action of probiotic intervention in metabolic and neuropsychiatric disorders have been progressively identified, including gut microbial alterations, intestinal inflammation, and intestinal permeability, neurons in the hippocampus, neuroinflammation, the HPA axis, the serotonergic system, and GABA, but none have been fully elucidated; (iv) Some probiotics (VSL#3) may be harmful to healthy people, even though these probiotics can be very therapeutic when consumed in disease states.

Overall, the current literature provides overwhelming evidence of the benefits of probiotics as supplements and their therapeutic potential for obesity-related cognitive impairment. Probiotic treatment modulates body weight and metabolic parameters (insulin sensitivity, inflammatory response, and energy metabolism) and can improve cognitive behavior. However, the gross generalization of these results should be interpreted with caution, as some studies have shown that some probiotics may be ineffective or may even exert a paradoxical effect. In addition, lack of consistency in study sample sizes, probiotic dosing parameters, treatment duration, and dosing methods hindered the possibility of conducting a comparative analysis between studies.

In the face of these existential problems, we believe that (i) more research should be done to explore the current knowledge on microbiota-host interactions and how probiotics can be used to regulate this dynamic relationship; (ii) Standardized clinical protocols for applying probiotic preparations should be developed, and research at the human level should be increased; (iii) Designing of probiotic functional foods that are highly viable, stress-resistant, and suitable for transport to improve microbial viability, stable colonization, and long-term dietary adherence must be carried out in the near future. Much work remains to ensure that probiotic therapies meet the medical application safety, purity, and efficacy standards. By further understanding the mechanisms of probiotic benefits and harm to host health, there is an opportunity to develop safe and targeted therapies that maximize the potential of probiotics in combating metabolic pathologies and neurological diseases.

## Author contributions

YC conceived and drafted the article. PL, XZ, and JY performed the literature search. QC critically revised the work. YC had primary responsibility for the final content. All authors read and approved the final manuscript. All authors contributed to the article.

## Glossary

**Table:**

AD	Alzheimer’s disease
AF	Abdominal fat
AKK	Akkermansia muciniphila
BAs	Bile acids
BBB	Blood-brain barrier
BDNF	Brain-derived neurotrophic factor
BMI	Body mass index
CCL2	C-C motif chemokine 2
CCO	Cytochrome c oxidase
CNS	Central nervous system
DAO	Diamine oxidase
DCA	Deoxycholic acid
D3-5	Lactobacillus paracasei D3-5 strain
FM	Fat mass
GABA	Gamma-aminobutyric acid
HFD	High-fat diets
HFHC	High-fat, high-cholesterol
HPA	Hypothalamic-pituitary-adrenal axis
HF	High-fat
5-HT	5-hydroxytryptamine
IL	Interleukin
LPS	Lipopolysaccharides
LPJBC5	Lactobacillus plantarum JBC5
LGG	Lacticaseibacillus rhamnosus
LTA	Lipoteichoic acid
LBP	Lipopolysaccharide-binding protein
mRNA	Messenger RNA
MAPK	Mitogen-activated protein kinase
MMS	Metabolic-mood-syndrome
MCP-1	Monocyte chemoattractant protein-1
NF-κB	Nuclear factor-κB
NAFLD	Non-alcoholic fatty liver disease
tOK67	tyndallized OK67
TNF-α	Tumor necrosis factor-α
TLRs	Toll-like receptors
WHR	Waist-to-hip
